# Supplementary material for: Sclerostin antibody improves alveolar bone quality in the Hyp mouse model of X-linked hypophosphatemia (XLH)
Source: Int J Oral Sci. 2023 Oct 10;15:47. doi: 10.1038/s41368-023-00252-1 (PMC10562382; doi:10.1038/s41368-023-00252-1)
Supplement: Supplementary file 1 — Supplementary Materials [file 41368_2023_252_MOESM1_ESM.docx]

**Sclerostin antibody improves alveolar bone quality the *Hyp* mouse model of XLH**

Kelsey A. Carpenter^1^, Delia O. Alkhatib^1^, Bryan A. Dulion^1^, Elizabeth Guirado^2^, Shreya Patel^1^, Yinghua Chen^2^, Anne George^2^, Ryan D. Ross^1,3,4^

1. Department of Anatomy & Cell Biology, Rush University Medical Center, Chicago, IL
2. Department of Oral Biology, The University of Illinois at Chicago, Chicago, IL
3. Department of Orthopedic Surgery, Rush University Medical Center, Chicago, IL
4. Department of Microbial Pathogens and Immunity, Rush University Medical Center, Chicago, IL

**Supplemental Materials**


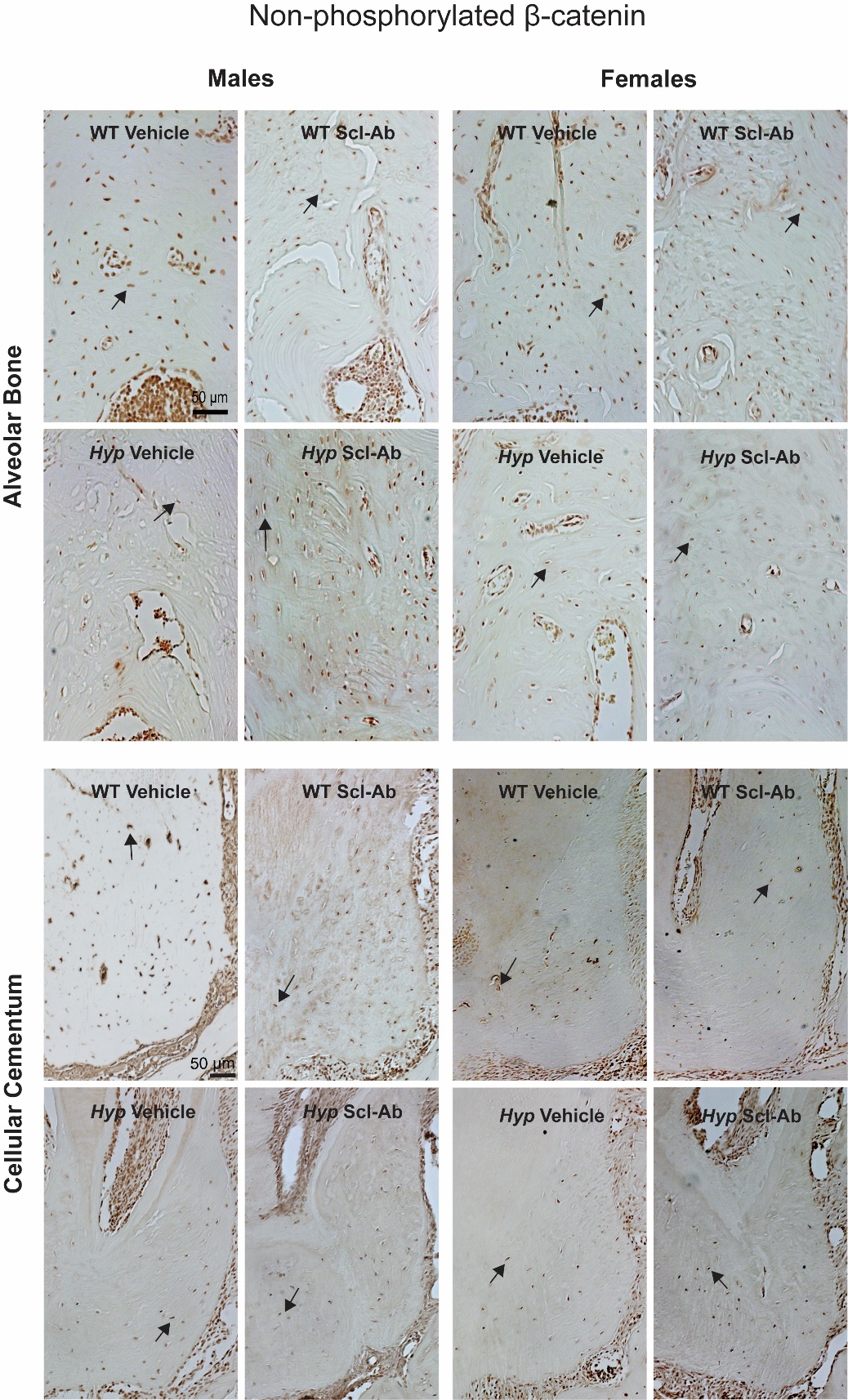


**Supplemental Figure 1:** Non-phosphorylated β-catenin immunostaining of alveolar bone and cellular cementum in male (left) and female (right) WT and Hyp mice treated with vehicle or Scl-Ab. Alveolar bone was evaluated between the tooth roots of the first molar (M1), while the cellular cementum was evaluated in the mesial root of M1. Arrows point to positively stained cells.

**
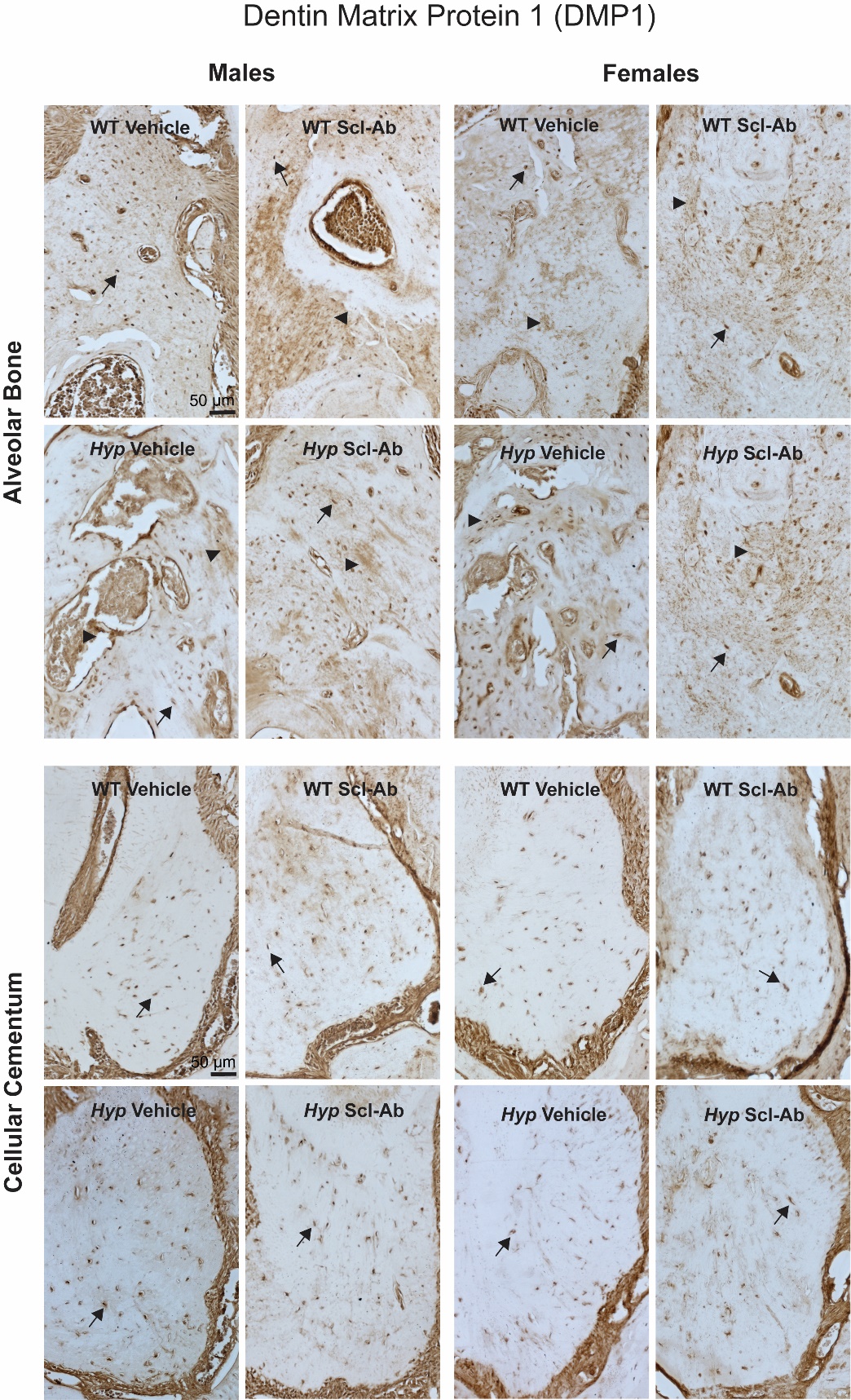
**

**Supplemental Figure 2:** DMP1 immunostaining of alveolar bone and cellular cementum in male (left) and female (right) WT and Hyp mice treated with vehicle or Scl-Ab. Alveolar bone was evaluated between the tooth roots of the first molar (M1), while the cellular cementum was evaluated in the mesial root of M1. Arrows point to positively stained cells.


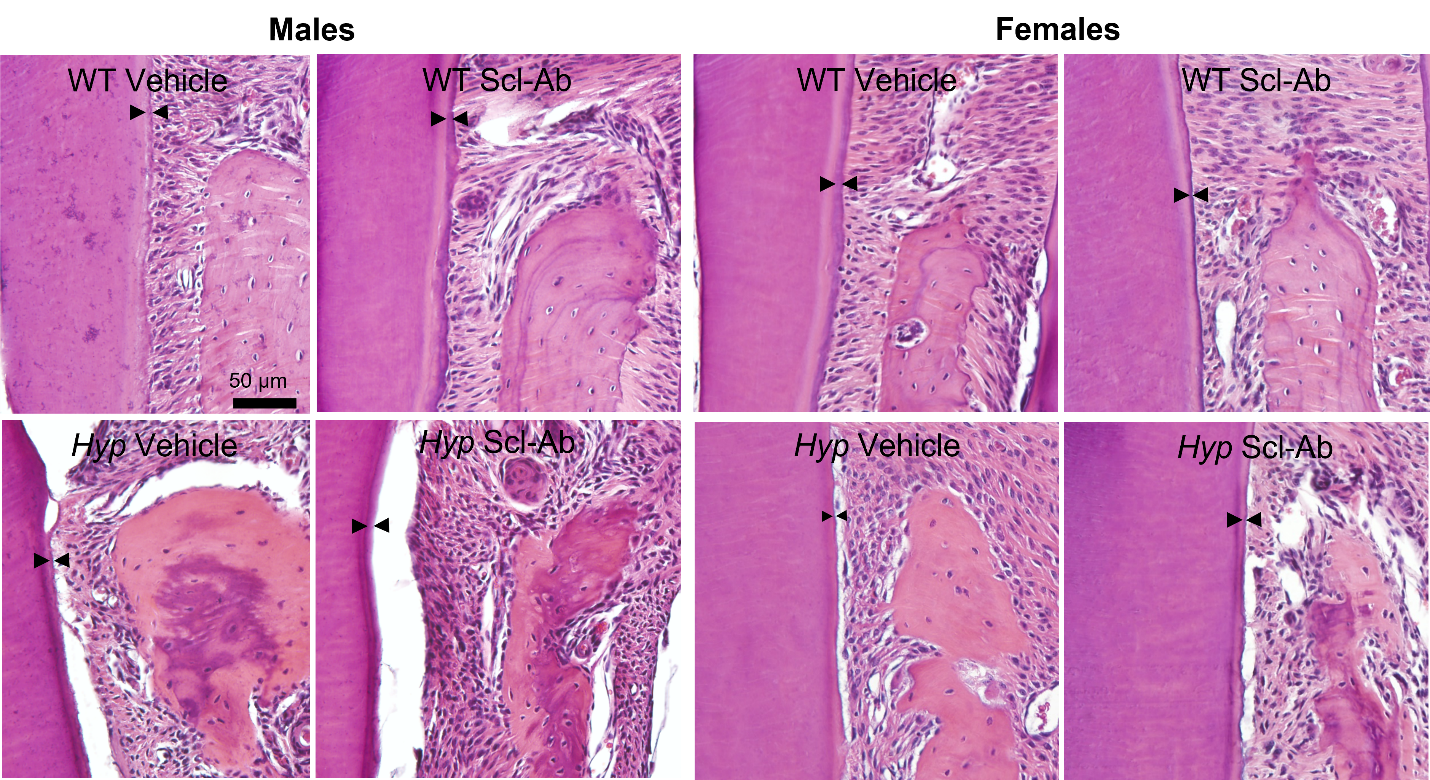


**Supplemental Figure 3:** Representative H&E stained saggital sections of acellular cementum on the distal root of the first molar (indicated between arrowheads) from male (left) and female (right) WT and *Hyp* animals treated with vehicle or Scl-Ab.


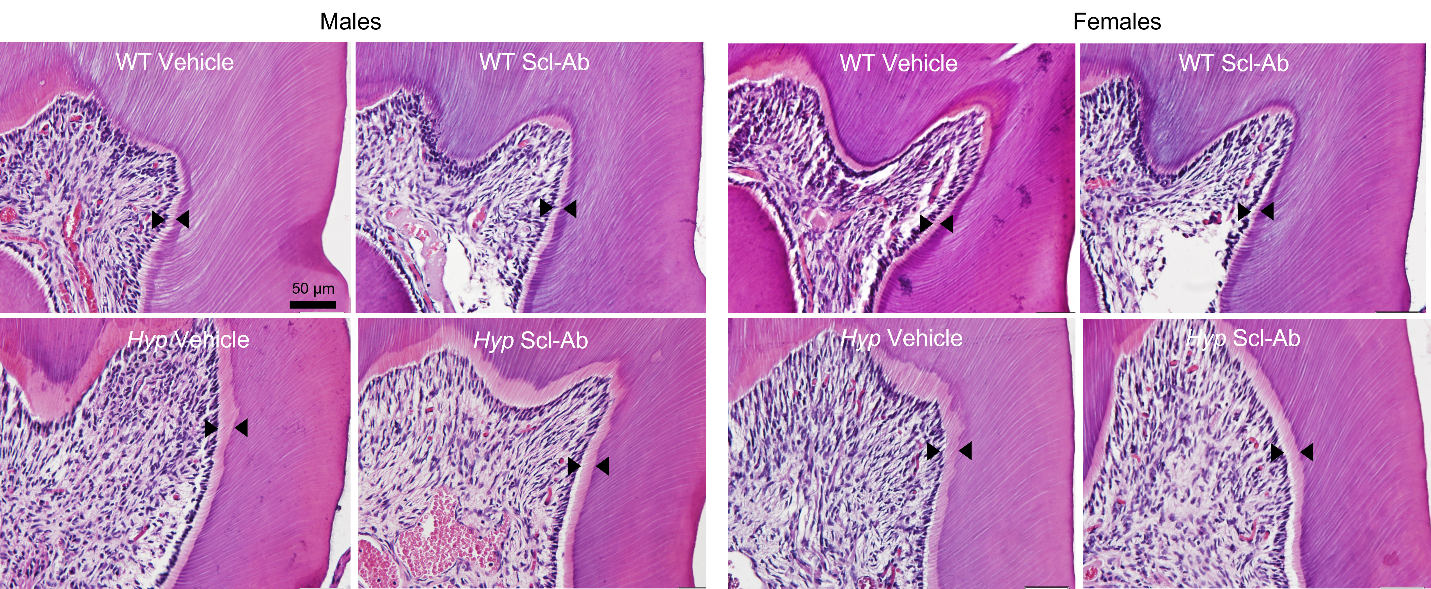


**Supplemental Figure 4:** Representative H&E-stained saggital sections of pre-dentin (indicated between arrowheads) from male (left) and female (right) WT and *Hyp* animals treated with vehicle or Scl-Ab.


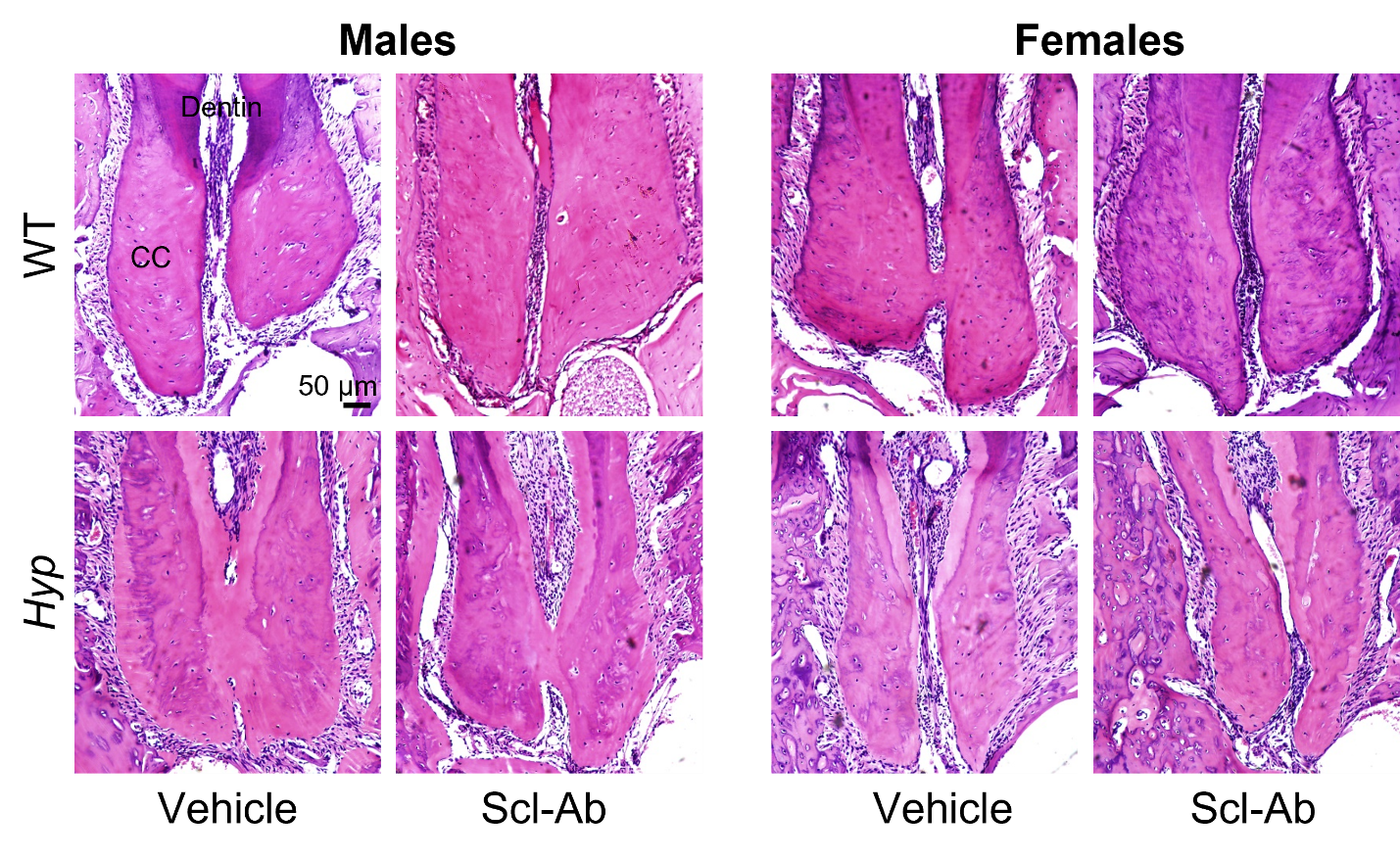


**Supplemental Figure 5:** Representative H&E-stained coronal sections of cellular cementum on the mesial root of the first molar from male (left) and female (right) WT and *Hyp* animals treated with vehicle or Scl-Ab. Dentin and cellular cementum (CC) are labeled for clarity.


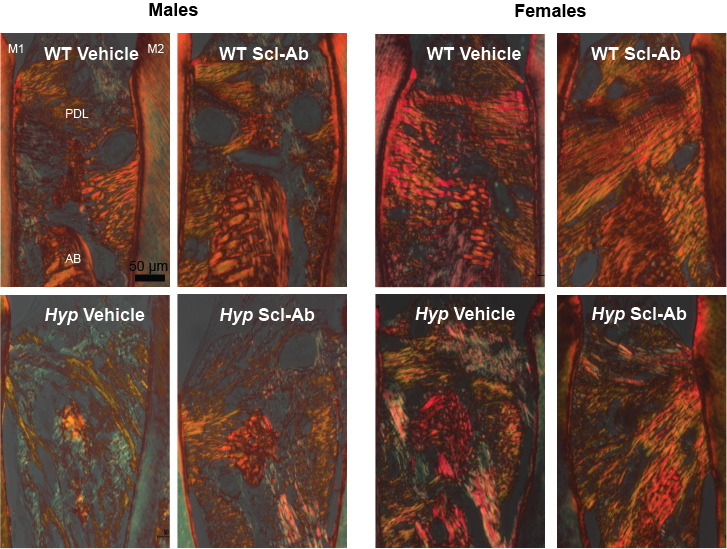


**Supplemental Figure 6:** Representative polarized picrosirius red stained periodontal ligament (PDL) in sagittal mandibular sections from male (left) and female (right) WT and *Hyp* animals treated with vehicle or Scl-Ab. The root surfaces of the first (M1) and second (M2) molar and the alveolar bone (AB) are marked for orientation.

**Supplemental Table 1:** Micro-computed tomography results from dentoalveolar tissues.

| Variable | WT Vehicle | WT Scl-Ab | *Hyp* Vehicle | *Hyp* Scl-Ab | Genotype | Treatment | Interaction |
| --- | --- | --- | --- | --- | --- | --- | --- |
| **Males:** | | | | | | | |
| Alveolar bone volume per total volume (AB BV, TV) | 0.58 ± 0.04 | 0.87 ± 0.02**^a^** | 0.36 ± 0.05**^b^** | 0.52 ± 0.04**^a,b^** | **<0.001** | **<0.001** | **<0.001** |
| Alveolar bone volume (mm^3^) | 0.15 ± 0.01 | 0.23 ± 0.02**^a^** | 0.12 ± 0.02**^b^** | 0.17 ± 0.03**^a^** | **<0.001** | **<0.001** | **0.016** |
| Total volume (mm^3^) | 0.26 ± 0.03 | 0.27 ± 0.02 | 0.33 ± 0.03**^b^** | 0.32 ± 0.03**^b^** | **<0.001** | 0.890 | **0.702** |
| Alveolar tissue material density (AB TMD, mg HA/mm^3^) | 1105 ± 18.69 | 1153 ± 21.02**^a^** | 946 ± 17.61**^b^** | 954 ± 18.06**^b^** | **<0.001** | **<0.001** | **0.004** |
| Enamel volume (mm^3^) | 0.12 ± 0.01 | 0.13 ± 0.01 | 0.11 ± 0.01**^b^** | 0.11 ± 0.01**^b^** | **<0.001** | 0.745 | 0.394 |
| Enamel tissue mineral density (mg HA/ mm^3^) | 1938 ± 34.7 | 1943 ± 26.4 | 1958 ± 40.5 | 1943 ± 31.6 | 0.328 | 0.624 | 0.347 |
| Cementum volume (mm^3^) | 0.25 ± 0.02 | 0.26 ± 0.03 | 0.24 ± 0.02 | 0.25 ± 0.02 | 0.245 | 0.281 | 0.522 |
| Cementum tissue mineral density (mg HA/mm^3^) | 880 ± 6.15 | 872 ± 5.53^a^ | 869 ± 5.60^b^ | 864 ± 6.84^b^ | **<0.001** | **0.001** | 0.400 |
| Dentin volume (mm^3^) | 0.71 ± 0.03 | 0.71 ± 0.05 | 0.53 ± 0.03^b^ | 0.51 ± 0.02^b^ | **<0.001** | 0.351 | 0.705 |
| Dentin tissue mineral density (mg HA/mm^3^) | 1238 ± 15.93 | 1240 ± 15.67 | 1244 ± 18.72 | 1237 ± 16.48 | 0.774 | 0.631 | 0.449 |
| Pulp volume (mm^3^) | 0.035 ± 0.003 | 0.036 ± 0.003 | 0.046 ± 0.005**^b^** | 0.047 ± 0.003**^b^** | **<0.001** | 0.381 | 0.920 |
| **Females:** | | | | | | | |
| Alveolar bone volume per total volume (AB BV, TV) | 0.73 ± 0.06 | 0.91 ± 0.06**^a^** | 0.59 ± 0.10**^b^** | 0.66 ± 0.04 **^a^** | **<0.001** | **<0.001** | **0.026** |
| Alveolar bone volume (mm^3^) | 0.16 ± 0.03 | 0.25 ± 0.03**^a^** | 0.16 ± 0.03**^b^** | 0.17 ± 0.04 | **<0.001** | **<0.001** | 0.096 |
| Total volume (mm^3^) | 0.62 ± 0.03 | 0.27 ± 0.03 | 0.27 ± 0.04**^b^** | 0.26 ± 0.05 | 0.954 | 0.850 | 0.620 |
| Alveolar tissue material density (AB TMD, mg HA/mm^3^) | 1123 ± 37.7 | 1153 ± 42.1 | 1023 ± 48.3**^b^** | 1012 ± 36.8**^b^** | **<0.001** | 0.435 | 0.109 |
| Enamel volume (mm^3^) | 0.13 ± 0.02 | 0.13 ± 0.02 | 0.11 ± 0.01**^b^** | 0.11 ± 0.01**^b^** | **0.001** | 0.850 | 0.845 |
| Enamel tissue mineral density (mg HA/ mm^3^) | 1951 ± 22.9 | 1952 ± 43.9 | 1945 ± 31.4 | 1936 ± 38.6 | 0.309 | 0.684 | 0.627 |
| Cementum volume (mm^3^) | 0.25 ± 0.03 | 0.26 ± 0.02 | 0.25 ± 0.03 | 0.24 ± 0.03 | 0.184 | 0.935 | 0.239 |
| Cementum tissue mineral density (mg HA/mm^3^) | 878 ± 5.76 | 871 ± 6.20^a^ | 873 ± 4.30 | 874 ± 12.98 | 0.698 | 0.217 | 0.125 |
| Dentin volume (mm^3^) | 0.70 ± 0.06 | 0.70 ± 0.04 | 0.55 ± 0.05^b^ | 0.53 ± 0.08^b^ | **<0.001** | 0.505 | 0.537 |
| Dentin tissue mineral density (mg HA/mm^3^) | 1247 ± 14.17 | 1242 ± 20.11 | 1242 ± 19.04 | 1237 ± 20.54 | 0.392 | 0.406 | 0.931 |
| Pulp volume (mm^3^) | 0.036 ± 0.004 | 0.038 ± 0.003 | 0.044 ± 0.005**^b^** | 0.042 ± 0.004**^b^** | **<0.001** | 0.857 | **0.043** |
| The sample size for microCT parameters are as follows:  n=10, 10, 10, 9 for male WT+vehicle, WT+Scl-Ab, Hyp+vehicle, Hyp+Scl-Ab and n=13, 13, 13, 8 for female WT+vehicle, WT+Scl-Ab, Hyp+vehicle, Hyp+Scl-Ab  ^a^ Indicates significant differences between vehicle and Scl-Ab treated mice of the same genotype.  ^b^ Indicates significant differences from vehicle treated WT mice. | | | | | | | |
